# A labeling strategy for the three-dimensional recognition and analysis of microvascular obstruction in ischemic stroke

**DOI:** 10.7150/thno.76879

**Published:** 2023-01-01

**Authors:** Yusha Li, Jianyi Xu, Tingting Yu, Jingtan Zhu, Ang Xuan, Xiaomei Liu, Pingfu Wang, Dongyu Li, Dan Zhu

**Affiliations:** 1 Britton Chance Center for Biomedical Photonics - MoE Key Laboratory for Biomedical Photonics, Wuhan National Laboratory for Optoelectronics - Advanced Biomedical Imaging Facility, Huazhong University of Science and Technology, Wuhan, Hubei 430074, China.; 2 Optics Valley Laboratory, Hubei 430074, China.

**Keywords:** microvascular obstruction, three-dimensional recognition, no-reflow, vascular lumen narrowing, neutrophil

## Abstract

**Rationale:** Large vessel recanalization in ischemic stroke does not always go along with tissue reperfusion, a phenomenon called “no-reflow”. However, knowledge of the mechanism of no-reflow is limited because identifying microvascular obstruction across the cortex and subcortex both in clinical and experimental models is challenging. In this study, we developed a smart three-dimensional recognition pipeline for microvascular obstruction during post-ischemia reperfusion to examine the underlying mechanism of no-reflow.

**Methods:** Transient (60 min) occlusion of the middle cerebral artery (tMCAo) in mice was induced using a filament. Two different fluorophore-conjugated tomato lectins were injected into mice via the tail vein before and after ischemia/reperfusion (I/R), respectively, one to label all blood vessels and the other to label functional blood vessels. Post-I/R microvascular obstruction was visualized using combined iDISCO+-based tissue clearing and optical imaging. Arterioles and capillaries were distinguished using whole-mount immunolabeling with an anti-αSMA antibody. Circulating neutrophils were depleted utilizing an anti-Ly6G antibody. Brain slices were immunostained with the anti-Ly6G antibody to identify co-localized blockage points and neutrophils. MATLAB software was used to quantify the capillary diameters in the ipsilateral brain from the normal and tMCAo mice.

**Results:** Microcirculatory reperfusion deficit worsened over time after I/R. Microvascular obstruction occurred not only in arterioles but also in capillaries, with capillary obstruction associated with local capillary lumen narrowing. In addition, the depletion of circulating neutrophils mitigated reperfusion deficit to a large extent after I/R. The co-localization of blockage points and neutrophils revealed that some neutrophils plugged capillaries with coexisting capillary lumen narrowing and that no neutrophil was trapped in heaps of blockage points. Quantification of the capillary diameter showed that capillary lumen shrunk after I/R but returned to typical measurements when intravascular neutrophils were depleted.

**Conclusions:** According to our findings, both vascular lumen narrowing and neutrophil trapping in cerebral microcirculation are the key causes of microvascular obstruction after I/R. Also, the primary contribution by neutrophils to microvascular obstruction does not occur through microemboli plugging but rather via the exacerbation of capillary lumen narrowing. Our proposed method will help monitor microcirculatory reperfusion deficit, explore the mechanism of no-reflow, and evaluate the curative effect of drugs targeting no-reflow.

## Introduction

Ischemic stroke, caused by the occlusion or stenosis of the artery, remains the second highest cause of death and a leading cause of disability worldwide 1. Recent progress in ischemic stroke treatment, including advances in intravenous thrombolysis and mechanical thrombectomy, has improved clinical outcomes remarkably 2, 3. However, poor clinical outcomes continue to occur even after successful recanalization therapies and have been attributed to hemorrhagic transformation, reperfusion injury, and incomplete microcirculatory reperfusion 4, 5.

Microcirculatory reperfusion failure, known as the “no-reflow” phenomenon, not only affects tissue survival but also hinders drug delivery to tissues for neuroprotection 6. The no-reflow phenomenon involving microvascular obstruction in stroke has been credited to vasoconstriction and microthrombus during post-ischemia reperfusion 7. A previous report suggested that endothelial and perivascular glial swelling result in a decrease in luminal size after ischemia/reperfusion (I/R), impeding blood flow 8. More recent studies have shown that an ischemia-induced continuous contraction of vascular mural cells causes blood cell entrapment after reperfusion 9-11. Post-ischemia microcirculatory thrombi contain erythrocytes, degranulated platelets, and polymorphonuclear leukocytes 12-14. The plugging of brain capillaries by neutrophils is considered a major cause of reperfusion deficit in a stroke 15, 16. However, despite these proposed factors, the mechanisms leading to microcirculatory reperfusion disturbances have yet to be fully elucidated, and effective therapies for improving capillary flow after recanalization remain to be established 17.

In order to examine these problems, the microvascular obstruction involved in no-reflow must be identified. Various *in vivo* optical microscopes have been used to continuously monitor the dynamic process of a microcirculatory change but only in the cerebral cortex. One investigation performed transcardiac dye perfusion on animal models and found ischemia-induced microcirculatory reperfusion failure in thin slices 8. However, the investigation could neither accurately identify occluded blood vessels nor reconstruct the vascular network to establish connectivity. In general, there is still no three-dimensional (3D) method for visualizing occluded microvessels across the cerebral cortex and subcortex during post-ischemia reperfusion. Combining tissue optical clearing with optical imaging has provided a powerful tool for 3D visualization of microvessels in brain blocks or whole brains using fluorescence labeling 18, 19.

In this work, we proposed a smart labeling strategy for identifying microvascular obstruction in mice by injecting the mice with two different fluorophore-conjugated tomato lectins before and after I/R, respectively. The tissue clearing technique and optical imaging were combinedly used to visualize 3D occluded blood vessels and observe the development of microcirculatory obstruction over time. We determined the location of blockage points using whole-mount immunolabeling with an anti-αSMA antibody and investigated the source of capillary obstruction. To further elucidate the mechanisms of no-reflow, we also examined whether and how neutrophils promote capillary flow stagnation using an anti-Ly6G antibody.

## Methods

### Animals

Animal experiments strictly complied with the Experimental Animal Management Ordinance of Hubei Province, China, and were approved by the Institutional Animal Ethics Committee of Huazhong University of Science and Technology. Two to three months old male C57Bl/6j mice were used in all experiments. The mice had free access to water and food and were housed in a room with a 12-hour light/dark cycle.

### Ischemic stroke model

Transient (60 min) occlusion of the middle cerebral artery (tMCAo) in mice was carried out using an intraluminal filament technique described previously 20. Briefly, mice were first anesthetized and maintained with 3% and 1.5% isoflurane, respectively, in the presence of the continuous supply of 300 mL/min air. An incision was performed at the midline of the neck for a blunt dissection to expose the trachea. The left common, external, and internal carotid arteries (CCA, ECA, and ICA) were carefully dissected from surrounding tissues, with the ECA permanently ligatured and the CCA and ICA temporarily ligatured with a 6-0 silk suture. A silicone-coated nylon monofilament (602256PK5Re, Doccol Corporation, Sharon, USA) was inserted through a small cut in the ECA, then into the ICA, and pushed until reaching the origin of the MCA in the Willis circle. The monofilament was left in place for 1 h of ischemia and then withdrawn to restore MCA recanalization for reperfusion. The body temperature of mice was kept constant at 37 °C during the surgical procedure using a temperature-controlled heating pad.

### Experimental groups

A total of 75 mice were used for this study, including 4 of which died and 5 of which were excluded because they did not show enough reduction in cortical blood flow after occlusion of the MCA or enough restoration of cortical blood flow after recanalization of the MCA. To assess the development of microvascular obstruction over time, the stroke model mice were randomly subjected to 0.5 h (n = 6), 2 h (n = 9), or 6 h (n = 9) of reperfusion. To examine the contribution of neutrophils to no-reflow, mice were randomly allocated to the normal (n = 5), sham (n = 5), isotype (n = 7), and anti-Ly6G (n = 5) groups. In addition, 5 mice were allocated for 3D immunostaining to determine the location of blockage points, 5 mice were reserved for 2D immunostaining to evaluate the behavior of neutrophils and platelets involved in no-reflow, and 10 mice were booked for the verification of the accuracy of vascular labeling with tomato lectin in stroke-injured tissues.

For the normal group, no surgical procedures were performed; for the sham group, mice underwent the same anesthesia and surgical procedures as the other two experimental groups but not the MCA occlusion; for the isotype and anti-Ly6G antibody groups, mice were put through surgical procedures of the tMCAo model, i.e., 1 h of ischemia and 2 h of reperfusion. Additionally, mice in the isotype and anti-Ly6G antibody groups respectively received injections of 1 mg/kg bodyweight of the isotype control antibody (400544, Biolegend) and 1 mg/kg bodyweight of purified anti-Ly6G antibody (127632, Biolegend) through their tail veins 24 h and 12 h before surgery on the tMCAo model. All mice from the different groups were kept in cages in a randomized fashion; the researcher who performed surgery on the tMCAo model was blinded to the groups.

### Vascular labeling

DyLight 488, DyLight 594 and Dylight 649-conjugated *lycopersicon esculentum* (Tomato) lectin (LEL, DL-1174, DL-1177, DL-1178; Vector Laboratories) were used for vascular labeling. After the anesthesia with isoflurane, each mouse tail was placed in a beaker filled with warm water at 40 °C for 30 s to dilate the tail vein. 100-μl tomato lectin was then slowly injected into the tail vein using a 29-G needle.

**Figure 1:**
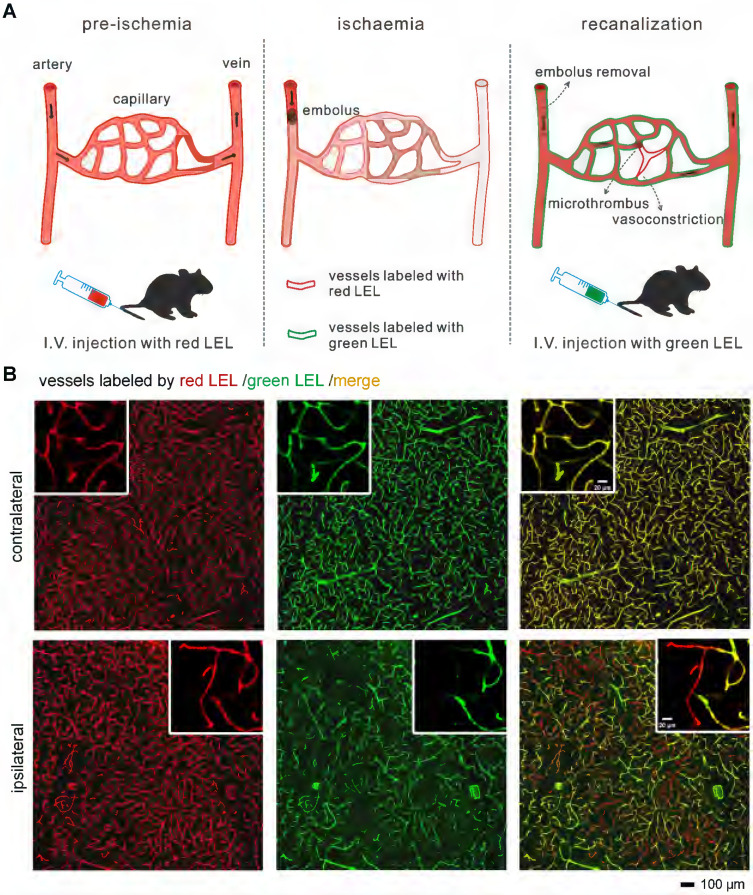
** Recognition of occluded microvessels after I/R** (**A**) The illustration of the method for recognizing occluded microvessels after I/R. Red and green LEL were respectively injected through the tail vein before ischemia (pre-ischemia) and after reperfusion (recanalization). Occluded microvessels were only labeled by red LEL and not green LEL. LEL: *lycopersicon esculentum* (tomato) lectin. (**B**) Representative fluorescence images of ipsilateral and contralateral microvessels from 100-μm tMCAo model mice brain slices. Mice were subjected to 1 h of ischemia and 2 h of reperfusion.

For recognition of microvascular obstruction after ischemia/reperfusion (I/R), two different fluorophore-conjugated tomato lectins were utilized to label blood vessels at different times. As shown in the schematic diagram (**Figure 1A**), mice were injected with two tomato lectins through the tail vein before and after I/R, respectively. The first injection was given 5 min before surgery of tMCAo model mice to label all blood vessels, and the second injection was introduced 5 min before the mice were sacrificed to label functional blood vessels. Microvessels only labeled by the first tomato lectin (red LEL) and not by the second tomato lectin (green LEL) were defined as occluded microvessels.

An anti-Glut1 antibody (07-1401; Sigma-Aldrich) and an Alexa Fluor 649-conjugated anti-CD31 antibody (102416; BioLegend) were also employed to label all blood vessels and functional vessels. The staining with the anti-Glut1 antibody was performed on 100-μm brain slices. The anti-CD31 antibody (10 μg pre mouse) was injected at the same time as the second tomato lectin.

### Animal sacrifice and sample preparation

The mice were deeply anesthetized with 3% isoflurane until they were unresponsive to the tail pinch test. They were then euthanatized with a continuous supply of 300 ml/min CO_2_. After sacrifice, the skins of the mice were dissected from the neck to the snout, and their heads were cut off and placed in 4% (w/v) paraformaldehyde (PFA) in 0.01 M phosphate-buffered saline (PBS), at 4 °C overnight in preparation for the fixation step. The brains were dissected the next day and placed again in 4% PFA for 24 h, at 4 °C. Subsequently, the brains were washed 4-5 times with 0.01 M PBS, embedded in 4% (w/v) agarose, cut into 100-μm or 1-mm cross slices with a vibratome (VT 1200s, Leica, Germany), and kept at 4 °C before further processing.

### Tissue optical clearing

Sample clearing was conducted following the iDISCO+ clearing protocol, as previously described, with minimal modification 21. Briefly, PFA-fixed brain blocks were dehydrated with a series of methanol solutions at increasing concentrations (20 vol. %, 40 vol. %, 60 vol. %, 80 vol. % in dH_2_O) for 2 h/step, bleached in methanol containing 5% hydrogen peroxide (H_2_O_2_) for 2 h/step, and dehydrated with 100% methanol overnight. After dehydration, the samples were incubated with a solution containing 66% dichloromethane in methanol for 2 h to remove the lipids. The samples were ultimately cleared and stored in dibenzyl ether until imaging. Uncleared samples were treated at room temperature, except for bleaching with H_2_O_2_ at 4 °C, while cleared samples were stored at 4 °C to maintain fluorescence. For clearing of whole mouse brains, the processing time was extended to 24 h/step for dehydration, 6 h for bleaching, 24 h for lipid removal, and 24 h for refractive index matching.

Other clearing protocols, including CUBIC 22, PACT 23, uDISCO 24, and FDISCO 25, were performed as laid out in the original papers.

### Statistical analysis

**Figure 2:**
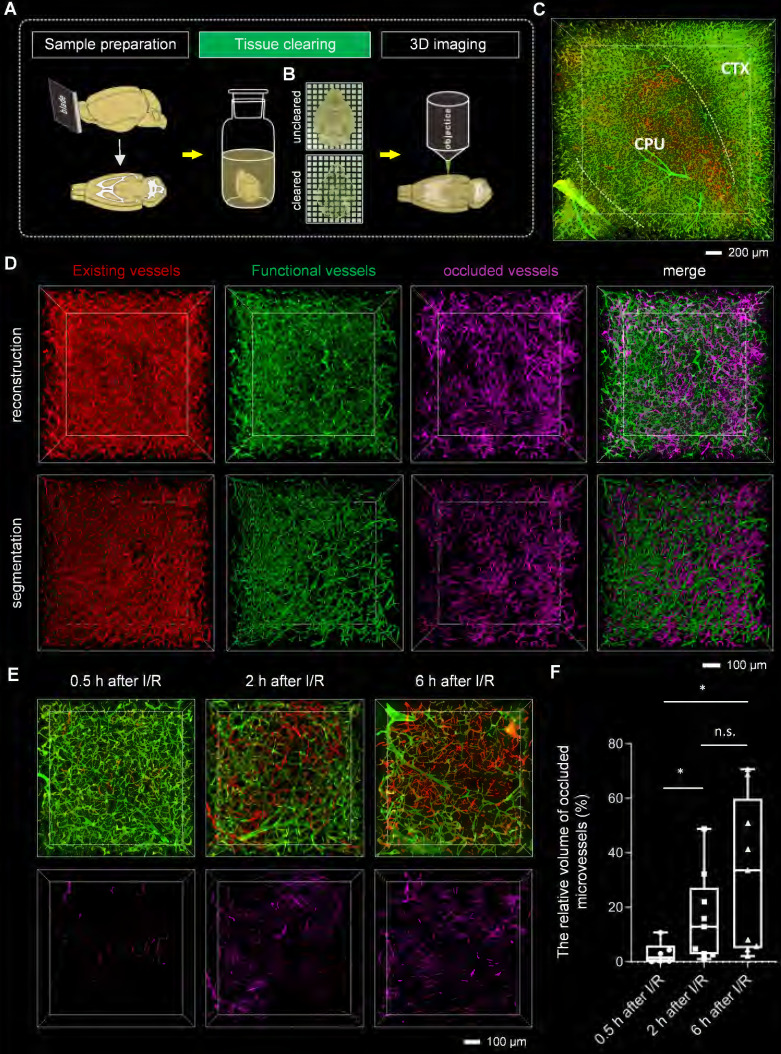
** Tissue optical clearing-assisted 3D analysis of microvascular obstruction after I/R** (**A**) Experimental workflow. (**B**) Bright-field images of the brain blocks before and after clearing with iDISCO+. Samples were placed in the air. Grid size, 1.44 mm × 1.44 mm. (**C**) 3D fluorescence images of microvessels in ischemic brain regions, including the cortex and striatum, of mice subjected to 1 h of ischemia and 2 h of reperfusion. CTX: cortex. CPU: caudate putamen (striatum). (**D**) Segmentation of microvessels using the “surface” tool of the Imaris software. (**E**) 3D visualization of occluded microvessels in the ipsilateral striatum at 0.5 h, 2 h, and 6 h after I/R. Red LEL was injected before surgery on the tMCAo model, and green LEL was injected 0.5 h, 2 h, or 6 h after withdrawing the filament. (**F**) A boxplot showing the relative volume of occluded microvessels at 0.5 h, 2 h, and 6 h after I/R (n = 6, 9, 9). Data represent medians (mix to max). n.s., p > 0.05; *p < 0.05; Brown-Forsythe and Welch ANOVA tests with post hoc two-stage linear step-up procedure of Benjamini, Krieger and Yekutieli for each comparison between groups.

Statistical analyses were carried out using the GraphPad Prism (version 8.0; GraphPad Software La Jolla, CA). The two-tailed t-test was used to compare data between two groups after testing for normality (Kolmogorov-Smirnov test) in **Figures S2B**, **S2D**, **S4E**, and **S7C**, and the Brown-Forsythe and Welch ANOVA tests were deployed for multiple comparisons between groups in **Figures 2F**,** S3D**, **S3F**,** 5D**,** 5E**, and **7C**. P values of less than 0.05 were considered statistically significant. All statistical tests and group sizes (n) are indicated in the figure legends.

Other detailed methods are available in the **Supplementary Materials**. All data and the supporting materials are available in the article and its **Supplementary Materials**.

## Results

### Recognition of microvascular obstruction after ischemia/reperfusion

In order to identify occluded microvessels *ex vivo*, we put forward an *in vivo* labeling strategy (**Figure 1A**). Investigating the effectiveness of this labeling strategy required establishing an animal model that can induce no-reflow. Therefore, a filament method for the transient (60 min) occlusion of the middle cerebral artery (tMCAo) in mice that can mimic the clinical scenario of ischemic stroke and mechanical thrombectomy was selected. Cortical hemodynamics were monitored using laser speckle contrast imaging before, during, and after tMCAo (**Figure S1A**).

The blood flow in cerebral MCA branches dropped to 17%±1% that of the baseline after the occlusion of the origin of the MCA but returned to 112%±21% that of the baseline after the filament was withdrawn (**Figure S1B**). Despite injecting mice with red and green LEL before and after tMCAo, some microvessels in the ipsilateral region only retained the red LEL label, appearing as red in the merged images. In contrast, microvessels in the contralateral region were completely labeled by two LEL colors and were shown as yellow in the merged images (**Figure 1B**). These findings confirmed that microvascular reperfusion in this model was not fully restored after recanalization of the occluded artery (MCA), and occluded microvessels can be recognized by labeling blood vessels with two different fluorophore-conjugated tomato lectins before and after ischemia/reperfusion (I/R), respectively.

Furthermore, we verified the accuracy of vascular labeling with tomato lectin in stroke-injured tissues using an anti-Glut1 antibody and an anti-CD31 antibody (**Figure S2**). The red LEL labeled all blood vessels in stroke-injured brain with that appeared identical to the labeling by the anti-Glut1 antibody through immunostaining. And the green LEL marked functional blood vessels in stroke-injured brain that looked like the labeling by the anti-CD31 antibody through intravenous injection.

### 3D visualization and analysis of microvascular obstruction after I/R

Tissue optical clearing techniques have provided powerful tools for the 3D analysis of microvessels. To analyze microvascular obstruction after I/R in 3D view, we employed the tissue clearing technique for imaging microvessels in thick brain blocks. To select a suitable clearing method, we cleared the tomato lectin-labeled brain blocks with several existing clearing methods, including CUBIC, PACT, uDISCO, FDISCO, and iDISCO+. We imaged the cleared brain blocks using an inverted confocal fluorescence microscope and quantified the fluorescence intensity of the blood vessels in the images (**Figures S3A-B**). The fluorescence signal intensities of the blood vessels in brain blocks cleared with solvent-based clearing methods, including iDISCO+, uDISCO, and FDISCO, were higher than those in brain blocks cleared with other methods. Furthermore, our comparison of transparent capacity and morphology maintenance of these three approaches (**Figures S3C-E**) revealed that the brain blocks cleared using iDISCO+ were almost colorless, less deformed, and had higher light transmittance than those cleared with the other two methods.

To evaluate the degree of microvascular obstruction after I/R, we combined iDISCO+ clearing with confocal microscopy imaging to capture microvessels in brain blocks (**Figures 2A-B**). We pictured ischemic cerebral microvessels labeled with two different fluorophore-conjugated tomato lectins in 3D view (**Figure 2C, Video S1**). In addition, by combining iDISCO+ clearing with light-sheet microscopy, we also achieved whole-brain imaging of microvessels in mice, enabling the examination of microvascular obstruction in various brain regions (**Figures S4A-B**, **Video S2**). In the ipsilateral brain region, sheets of microvessels labeled only with the red LEL (the injection before ischemia) but not with green LEL (the injection after reperfusion) were notable, namely “occluded microvessels” (**Figure 2C, Video S1 and S2**).

Red LEL and green LEL-labeled microvessels were respectively denoted as “existing vessels” and “functional vessels”. Microvessels were traced using the “filament tool” of the Imaris software, and the vascular length ratios of “existing vessels” to “functional vessels” were calculated to quantify the degree of microvascular obstruction after I/R (**Figure S4C, Video S3**). Results showed that some occluded microvessels were distributed in the ipsilateral striatum, but few were identified in the cortex (**Figure S4D**). There is proof that the MCA supplies blood to both the cortex and striatum of mice.^26^ We also found no perfusion after MCA occlusion in the cortex and striatum, suggesting that both are ischemic cores (**Figure S5**). The comparison of the degree of microvascular obstruction in the striatum and cortex revealed that it was more severe in the striatum than that in the cortex (~78% vs ~99%) (**Figure S4E**).

We further quantified the degree of microvascular obstruction via the relative vascular volume of occluded microvessels. Occluded microvessels were extracted, then reconstructed and analyzed (**Figures 2D and S6**). To explore the development of microvascular obstruction over time, occluded microvessels at 0.5 h/2 h/6 h after I/R were subtracted and segmented, and their relative vascular volumes were calculated. Per the findings, microvascular obstruction became severe gradually as reperfusion lengthened, from the initial blockage in downstream blood vessels to upstream blood vessels (**Figures 2E and 2F**).

### Capillary obstruction was associated with narrow capillary lumen after I/R

**Figure 3:**
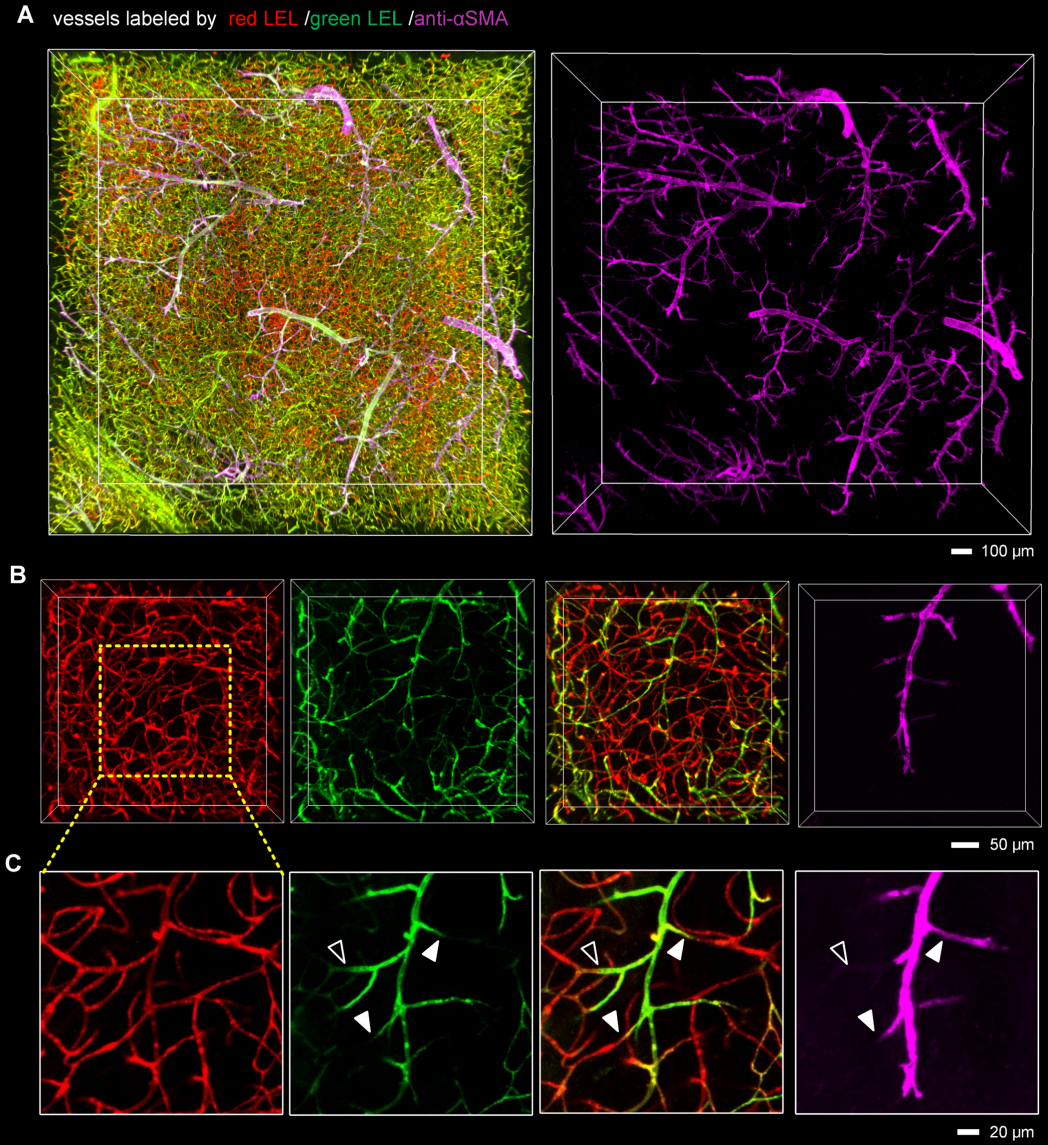
** The presence of blockage in the precapillary arterioles and capillaries** (**A**) 3D fluorescence images of microvessels in 1-mm brain slices immunostained with an anti-αSMA antibody, cleared with the iDISCO+ method, and imaged with confocal microscopy. Mice were injected with two different fluorophore-conjugated tomato lectins before and 2 h after tMCAo to identify microvascular obstruction. The anti-αSMA antibody was utilized to label arteries and arterioles. (**B, C**) Representative 3D reconstructed images (**B**) and MIP images (**C**) showing blockages in the arterioles and capillaries. The close arrowheads indicate blockage points located in the arterioles with αSMA labeling, and the open arrowhead shows the blockage point located in the capillary without αSMA labeling.

**Figure 4:**
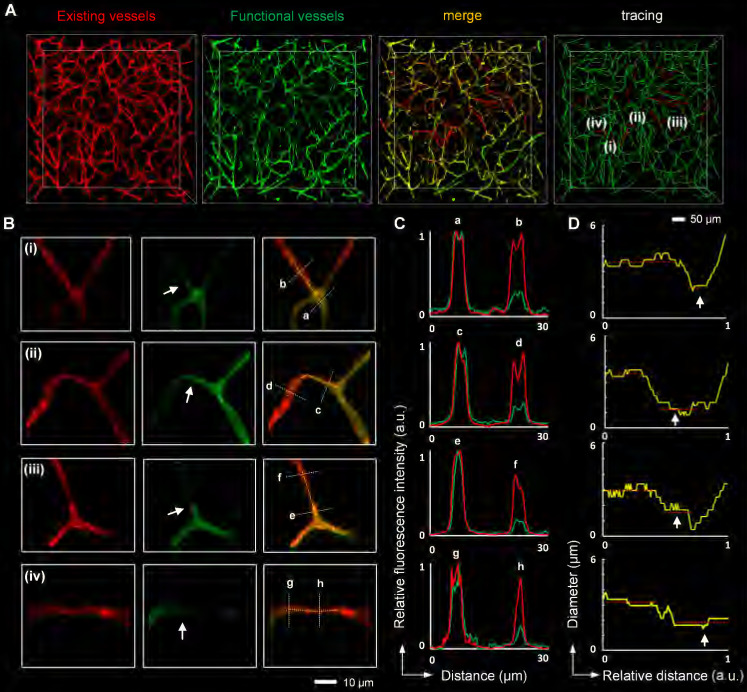
** Microconstriction observation in occluded capillaries after I/R** (**A, B**) High-resolution images of microvessels showing occluded capillaries (**A**) and microconstrictions in the vascular lumen (**B**). The images in (**B**) are cropped from that in (**A**). The white arrows in (**B**) indicate blockage points in the capillaries. (**C**) Normalized intensity profiles of the marked white lines (a-h) in (**B**). The red lines represent the signal from red LEL, and the green lines denote the signal from green LEL. (**D**) The graphic depiction of the diameters of the blood vessels marked with yellow lines in (**B**). The white arrows in (**D**) indicate reduced lumen diameters at the blockage points.

Since capillary hemodynamics is greatly affected by upstream arterioles, the location of the blockage point must be determined before the source of capillary obstruction can be identified. To accurately establish the location of the blockage, we conducted whole-mount immunolabeling using an anti-αSMA antibody to distinguish between arterioles and capillaries. Microvascular obstruction and the αSMA-labeled vasculature in the ipsilateral striatum were visualized in 3D view (**Figure 3A, Video S4**). The αSMA-labeled vasculature resembled perfect trees, with multiple short, straight branches protruding from the trunk. Upstream arteriole obstruction resulted in multiple downstream continuous microvessel flow stagnations (**Figure 3B**). And the blockage points were located not only in the arteriole with αSMA labeling but also in the capillary without αSMA labeling, suggesting that stalled capillary perfusion after I/R occurred not only as a consequence of increased precapillary arteriole tone but also due to the blockage in the capillary (**Figure 3C**). To ascertain the source of capillary obstruction, we imaged occluded capillaries using high magnification and quantified the diameter of the vascular lumen near blockage points (**Figure 4**). The outcome was the narrowing of the vascular lumen at the blockage points of occluded capillaries, suggesting that capillary obstruction after I/R was associated with microconstrictions in capillaries.

### Neutrophils promoted capillary flow stagnation after I/R

**Figure 5:**
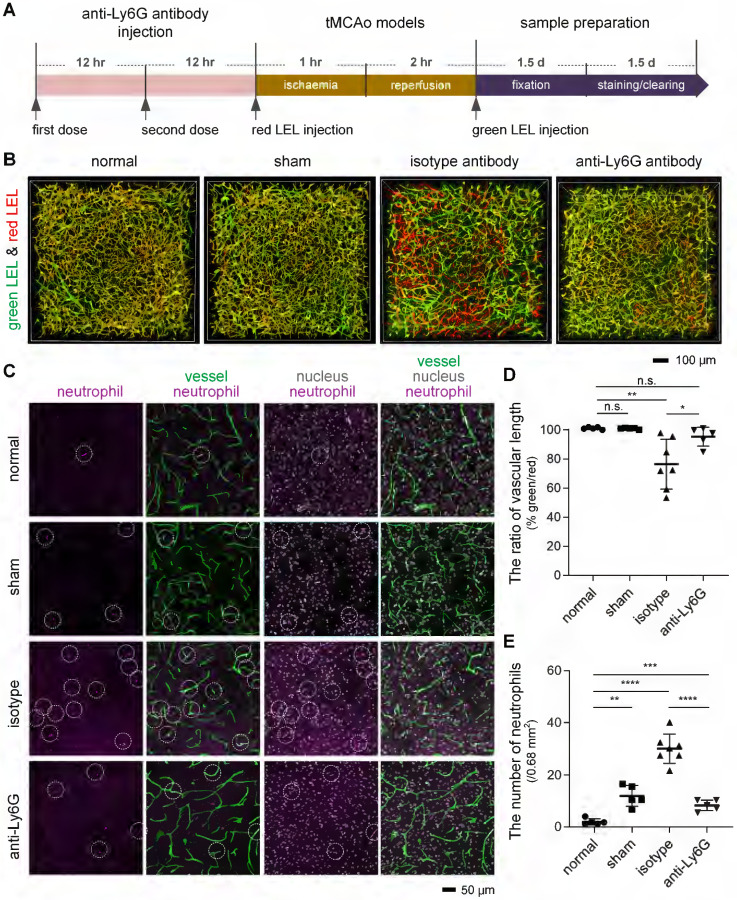
** Reduction in microvascular obstruction in the ischemic core and neutrophils in the cerebral microcirculation after I/R following treatment with an anti-Ly6G antibody** (**A**) The experimental flow showing the procedure and time. (**B**) 3D two-color fluorescence images of microvessels in cleared brain slices from the normal, sham, isotype, and anti-Ly6G groups. (**C**) Representative images of neutrophils in cerebral microcirculation from 100-μm brain slices from the normal, sham, isotype, and anti-Ly6G groups. The 100-μm brain slices were stained with an anti-Ly6G antibody. Intravascular markers containing anti-Ly6G (purple, neutrophils) and DAPI (blue, nucleus) signals were recorded as neutrophils (circled in white). (**D, E**) Quantification of the relative vascular length in the ipsilateral striatum (**D**) and the number of neutrophils in cerebral microcirculation (**E**) in mice from the normal, sham, isotype, and anti-Ly6G groups (n = 5, 5, 7, 5). Data represent the mean ± SD. n.s., p > 0.05; *p < 0.05; **p < 0.01; ***p < 0.001; ****p < 0.0001; Brown-Forsythe and Welch ANOVA tests with post hoc two-stage linear step-up procedure of Benjamini, Krieger and Yekutieli for each comparison between groups.

First, we tried to confirm the likelihood that neutrophils are responsible for microvascular obstruction after I/R. We applied a monoclonal anti-Ly6G antibody, which has been shown to target and deplete circulating neutrophils 27, before surgery on the tMCAo model (**Figure 5A**). Our assessment of the microvascular obstruction and intravascular neutrophils revealed that anti-Ly6G antibody treatment reduced occluded microvessels after I/R to a considerable degree, and this reduction was accompanied by a decrease in neutrophils in cerebral microcirculation (**Figures 5B-E, Video S5**). Quantitative analyses also established few neutrophils in mouse cerebral microcirculation under physiological conditions but significantly more neutrophil invasion of the cerebral microcirculation after I/R (**Figures 5C and 5E**). These results pointed to neutrophils as an important element promoting post-I/R microvascular obstruction. Additionally, neutrophils in contralateral cerebral microcirculation was identical in number to those in ipsilateral cerebral microcirculation (**Figure S7**), implying that neutrophil recruitment to an ischemic brain is not significant a few hours after I/R.

**Figure 6:**
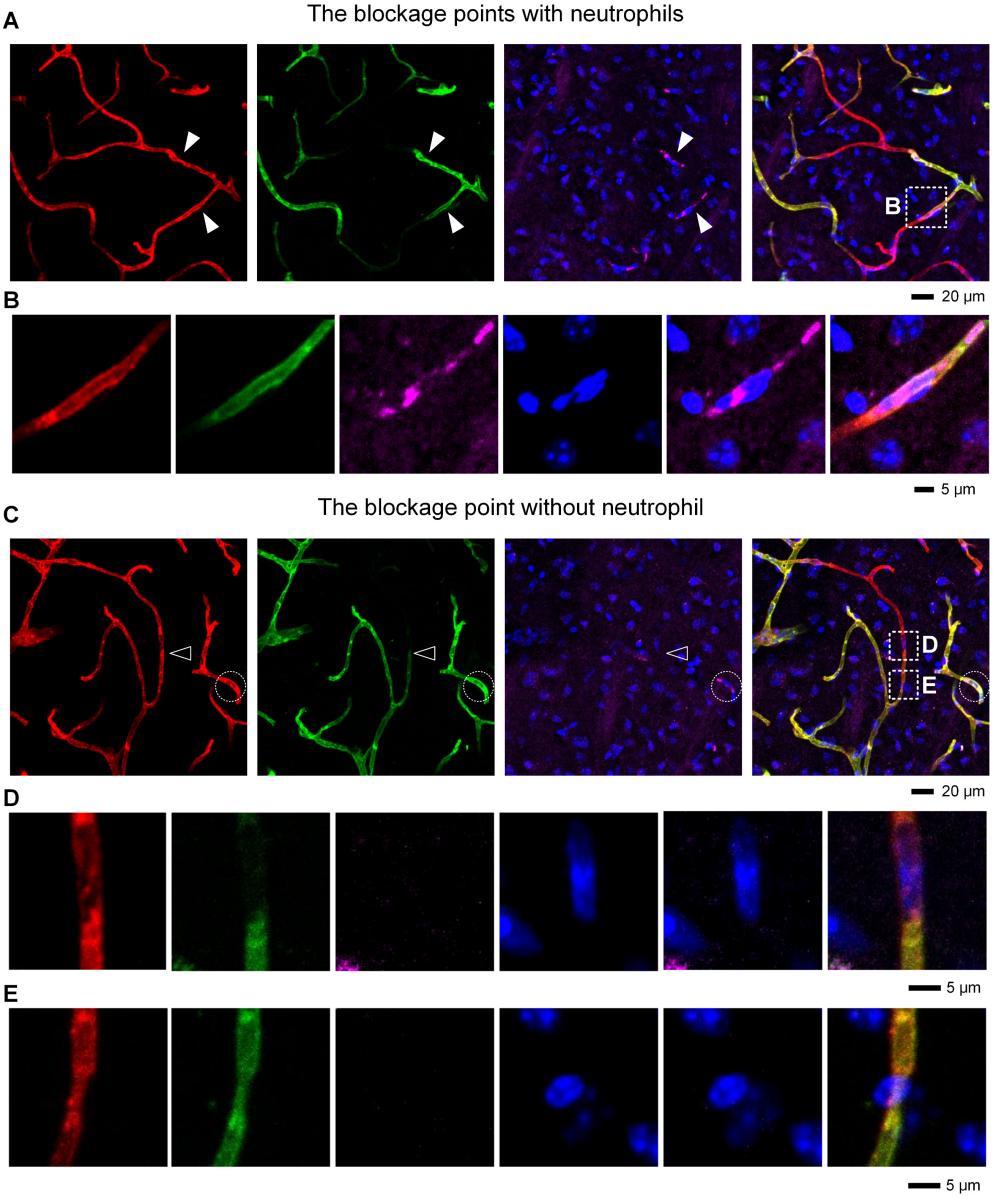
** Correlation between microvascular obstruction and neutrophil presence after I/R** (**A**) Representative images showing some capillaries plugged with neutrophils. The close arrowheads denote the blockage points with neutrophils. (**B**) Repeated images marked with boxes in (**A**). (**C**) Representative images showing the occlusion of some capillaries in the absence of neutrophils. The open arrowhead denotes the blockage point without neutrophils. The neutrophils are circled in the white circles. (**D, E**) Repeated images marked with boxes in (**C**). (**D**) The blockage point is void of neutrophils. The nucleus shown here is from an endothelial cell. (**E**) The co-localization of the narrow lumen of a vessel and the nucleus of a perivascular cell near the blockage points. The nucleus appears to lock the vessel like a claw. red, Existing vessels; green, Functional vessels; purple, Neutrophil; blue, Nucleus.

**Figure 7:**
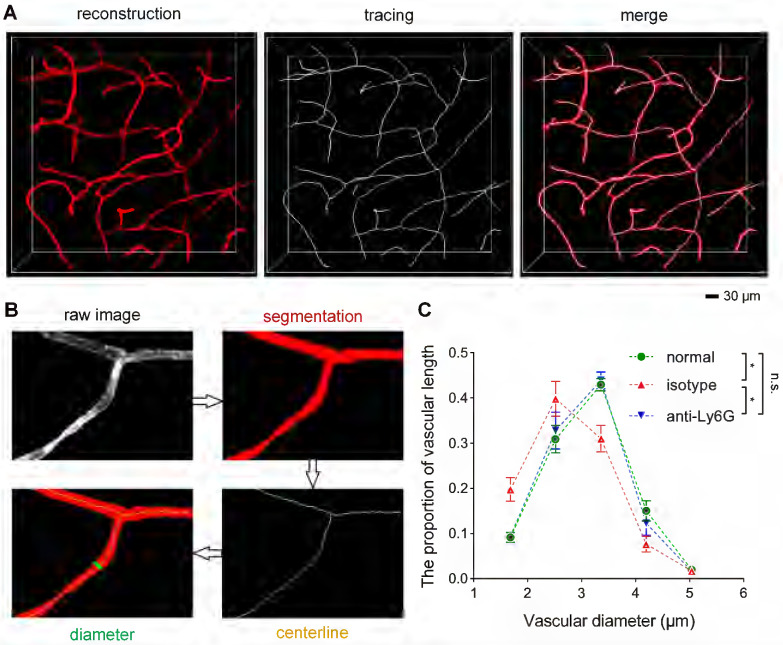
** Quantification of the vascular diameter** (**A**) 3D reconstruction and tracing of capillaries in cleared brain slices. Blood vessels were traced to quantify the vascular diameter using the MATLAB software. (**B**) Data processing for the calculation of the vascular diameter using the ImageJ and MATLAB software. (**C**) The frequency distribution of the ipsilateral striatal vascular diameter in the normal, isotype, and anti-Ly6G groups (n = 5). Data represent the mean ± SEM. n.s., p > 0.05; *p < 0.05; Brown-Forsythe and Welch ANOVA tests with post hoc Tamhane's T2 multiple comparisons test for each comparison between groups.

To understand how neutrophils contribute to microvascular obstruction, we further scrutinized the relative position of neutrophils to blockage points. Our results revealed that some capillaries were plugged with neutrophils, and the trapped neutrophils were compressed and denatured (**Figures 6A-B**). Some neutrophils were trapped behind narrowed regions in the occluded capillaries (**Figure S8A-B**), suggesting that neutrophils might plug capillaries with concurrent lumen narrowing. Numerous blockage points without neutrophils were also noted (**Figures 6C-D and S8C-E**). Our examination of the fraction of blockage points with and without neutrophils uncovered no neutrophils at 56 of the 78 blockage points in 5 mice, indicating that neutrophils plugging capillaries as microemboli was not the main path to capillary obstruction. Additionally, we found capillary lumen narrowing to be co-localized with claw-like nuclei of perivascular cells near the blockage points, signifying a correlation between capillary obstruction and the constriction of perivascular cells (**Figures 6E and S8B**). Capillary lumen diameters in the normal, isotype, and anti-Ly6G groups were also quantified (**Figure 7**). The frequency distribution of vascular diameters showed that capillary lumen diameters shrunk after I/R but returned to typical measurements when intravascular neutrophils were depleted using the anti-Ly6G antibody.

## Discussion

There is accumulating evidence that microcirculatory reperfusion failure occurs even after successful recanalization in ischemic stroke, potentially hampering the benefit of recanalization therapies 28. Here, we developed a method for visualizing occluded microvessels in 3D view after ischemia/reperfusion (I/R) to scrutinize the underlying mechanism of microcirculatory reperfusion failure.

### Our proposed strategy for evaluating microvascular obstruction

That large vessel recanalization does not always go along with tissue reperfusion in ischemic stroke has been attributed mainly to microvascular obstruction caused by vasoconstriction and microthrombus 17, 28. Previously, microvascular obstruction was identified by measuring hemodynamics *in vivo* using optical microscopy; however, in our investigation, we utilized *lycopersicon esculentum* agglutinin (tomato lectin) to identify microvascular obstruction after I/R. Tomato lectin, a common vascular-specific marker, has been used to label blood vessels through intravenous injection under physiological and pathological conditions. Since effective labeling is based on circulating tomato lectin, tomato lectin can be used to label functional blood flow vessels, thereby reflecting the perfusion status of the microcirculation. An additional tomato lectin conjugated with a different fluorophore also has to be injected into mice or used to stain brain samples to label all microvessels. Hence, combining vascular labeling via the use of two different fluorophore-conjugated tomato lectins can help determine microvascular obstruction after I/R. Previous studies also perfused the dye through the heart to examine the microvascular flow in ischemic stroke 8, 29; however, they were impossible to identify occluded microvessels. Our proposed strategy is also compatible with whole-mount immunostaining to identify cell types, enabling the examination of the vascular perfusion status and the differentiation between different sources of microcirculation stalling.

Several early observations indicated that no-reflow was occurred even when the microvascular bed was partially obstructed that permitted the flow of plasma and trapped the red blood cells 9, 11, 30. Our strategy identifies only occluded microvessels that block both the red blood cells and plasma, so it can detect no-reflow only when the damage become serious. Another limitation of our labeling strategy is blindness to the dynamics process of capillary blood flow. Existing evidence has demonstrated that the same capillary segments repeatedly stall and flow again in stroke or Alzheimer's disease 11, 27, 31. *In vivo* optical microscopy is often used to monitor the dynamic process of microvascular flow change 32, 33. For example, Erdener et al. detected dynamic flow stalling in cerebral capillaries using optical coherence tomography (OCT), and Robert et al. assessed blood flow regulation at various vascular sites using two-photon microscopy (TPM) 15, 16. However, OCT enables the quantitative volumetric imaging of dynamic blood flow, but it cannot differentiate between different sources of capillary stalling. TPM can differentiate between fluorescently-labeled cell structures, but its limited speed hampers its ability to continuously detect hemodynamics volumetrically. In general, our strategy can complement existing *in vivo* optical imaging methods to help evaluate microcirculation deficits in acute and chronic neurodegenerative conditions.

In this work, when evaluating the degree of microvascular obstruction, we used the Imaris software for quantification of vascular length and vascular volume. There are several newly reported tools that analyze multiple characteristic vascular network parameters, such as branching point and tortuosity, which can help us to conduct a more comprehensive 3D structural analysis of blood vessels in stroke 19, 34-36.

### Brain region differences in microvascular obstruction after I/R

We confirmed here that microvascular obstruction in a mouse model of ischemic stroke occurred after full recanalization of an occluded large artery. We only observed the incidence of occluded microvessels in the cortex and striatum, given that we had already noted individual variations in other ischemic brain regions in the stroke model we used in C57bl/6j mice 37. The microvascular obstruction in the striatum was more severe than that in the cortex. Previous studies found cerebral white matter to be inherently vulnerable to ischemia, possibly because the oligodendrocytes and myelin abundant in white matter are vulnerable to ischemia 38, 39. Zu-Lin Chen et al. also showed that astrocytes created relatively high signals with vascular mural cells in the striatum than in the cortex, rendering the striatum more susceptible to intracerebral hemorrhage 40. Based on these insights, we assumed that differences in cellular components and cellular contacts of brain regions possibly contribute to the difference in microcirculation changes between the striatum and cortex 41. The specific differences between the two brain regions that cause the disparity in the reperfusion deficit after I/R require further examination and should help assess the mechanism of no-reflow. Notably, we analyzed microvascular obstruction only in 1-mm brain blocks, but this pipeline also enables whole-brain imaging for the evaluation of various brain regions. Thus, while optical microscopy *in vivo* is limited to viewing the cerebral cortex through the cranial window, our proposed method could provide a useful tool for studying such differences between brain regions.

### The cell type responsible for capillary lumen narrowing in no-reflow

With our strategy, local changes in lumen diameter at capillary blockage points were easily discernable by assessing the vascular structure with high-resolution imaging. We have confirmed that microvascular obstruction after I/R is associated with vascular lumen narrowing. In the context of a stroke, a previous investigation demonstrated that vascular mural cell constriction in precapillary arterioles narrow vascular lumen and impede blood flow 11. However, whether capillary mural cells (pericytes) constrict and contribute to capillary obstruction remains debatable 42. It has been reported that smooth muscle actin, a contractile protein, is expressed in precapillary arteriole mural cells but is undetected in capillary pericytes in the mouse brain 11, 43. And capillary pericytes only partially cover the vessels, appearing as mesh-like or strand-like cells. Several studies assumed that the soma of capillary pericytes could exert tension on capillaries, resulting in local changes in capillary diameter 10, 42, 44, which is consistent with our finding that the nuclei of perivascular cells lock capillary lumens like claws. Early studies assumed that endothelial and perivascular glial swelling resulted in decreased luminal size; however, this assumption lacks direct evidence 8. Capillary-associated microglia have also been reported to regulate capillary vascular tone and play a more significant role in blood flow regulation than previously thought 45, 46.

Since capillary blood flow is greatly influenced by upstream arterioles, the location of the blockage point must be determined to accurately identify the cell type responsible for capillary lumen narrowing. However, it is difficult to determine the blockage point using* in vivo* optical microscopy 32. The protocol we have developed will be an indispensable tool in the future exploration of the ability of perivascular cells to control blood flow, which is critical to advancing therapies aimed at improving capillary flow after recanalization.

### Neutrophil behavior during no-reflow

Neutrophils, which are large and less deformable cells that move rather slowly in the microvasculature, are reportedly an aggravating element of no-reflow after I/R 12, 13, 15, 16. Researchers generally believe that neutrophils adhere to endothelial cells and become microemboli, plugging capillaries after ischemic stroke 47, 48. However, the physical impaction of neutrophils in narrow capillary lumens has also been proposed as a mechanism for microvascular obstruction during ischemia. And how neutrophils contribute to no-reflow after I/R remains unclear 17.

Employing antibody treatment targeting neutrophils, we revealed that reperfusion deficits were mitigated to a significant extent and capillary lumen diameters returned to typical measurements in tMCAo mice upon the depletion of neutrophils invading the brain. Previous studies only reported that neutrophils, as the major microemboli, plugged capillaries by adhering to the endothelium in the no-reflow phenomenon 15, 16. However, we hypothesize that neutrophils can exacerbate vascular lumen narrowing to promote microvascular obstruction after recanalization. It is possible that increased reactive oxygen species released from intravascular neutrophils combined with reduced NO released from damaged endothelial cells in stroke-injured brain drive pericyte constriction 9, 49.

Notably, platelets also aggregated in occluded microvessels, but only a few did so in flowing microvessels (**Figure S9**). There was little platelet aggregation at the site of blockage, so we speculated that platelets did not directly cause the blockage of blood vessels. Mohamad El Amki et al. recently reported that when using a thrombin model of stroke and thrombolysis, they noted platelet stalling in capillaries only on a few occasions 15. That the platelet-leukocyte-endothelial cell interactions in the cerebral microvasculature induced by I/R were implicated in no-reflow has been shown before 13. Given that the role of platelets involved in no-reflow remains unclear, our method is promising to help elucidate this issue. In the future, in-depth analyses should be conducted to establish clearer behavioral interpretations in relation to neutrophils and platelets in the no-reflow phenomenon in the brain; for example, how neutrophils trapped in cerebral microcirculation exacerbate vascular lumen narrowing in stroke-injured brain and whether neutrophils can impact no-reflow after I/R through interactions with platelets and endothelial cells.

## Conclusions

Here, we developed a pipeline for identifying and analyzing occluded blood vessels after ischemia/reperfusion (I/R) utilizing a vascular labeling strategy that employs two different fluorophore-conjugated lectins and a combination of tissue optical clearing and optical imaging. This pipeline revealed that microvascular obstruction worsened over time after I/R and was associated with vascular lumen narrowing and neutrophil invasion of cerebral microcirculation. The pipeline also showed that neutrophils do not contribute to microvascular obstruction primarily as microemboli after I/R, and they impact no-reflow by exacerbating vascular lumen narrowing. These findings help better understand reperfusion deficit after recanalization in ischemic stroke. We believe that this recognition strategy can catalyze more basic and clinical research broadly for recanalization therapy after ischemic stroke.

## Supplementary Material

Supplementary methods, figures and video legends.

Supplementary video 1.

Supplementary video 2.

Supplementary video 3.

Supplementary video 4.

Supplementary video 5.

## Abbreviations

I/R: ischemia/reperfusion
tMCAo: transient occlusion of middle cerebral artery
3D: three-dimensional
LEL: *lycopersicon esculentum* (tomato) lectin
OCT: optical coherence tomography
TPM: two-photon microscopy
