# Supplementary material for: A labeling strategy for the three-dimensional recognition and analysis of microvascular obstruction in ischemic stroke
Source: Theranostics. 2023 Jan 1;13(1):403–16. doi: 10.7150/thno.76879 (PMC9800741; doi:10.7150/thno.76879)
Supplement: Supplementary file 1 — Supplementary methods, figures and video legends. [file thnov13p0403s1.pdf]

Supplementary materials

A labeling strategy for the three-dimensional recognition and analysis of microvascular obstruction in ischaemic stroke

Yusha Li[1,2], Jianyi Xu[1,2], Tingting Yu[1,2], Jingtan Zhu[1,2], Ang Xuan[1,2], Xiaomei Liu[1,2], Pingfu Wang[1,2], Dongyu Li[1,2], Dan Zhu[1,2*]

[1]Britton Chance Center for Biomedical Photonics - MoE Key Laboratory for Biomedical Photonics, Wuhan National Laboratory for Optoelectronics - Advanced Biomedical Imaging Facility, Huazhong University of Science and Technology, Wuhan, Hubei 430074, China; [2]Optics Valley Laboratory, Hubei 430074, China.

*Corresponding author: Dan Zhu, Email: dawnzh@mail.hust.edu.cn

Supplementary methods

Laser Speckle Contrast Imaging (LSCI)

Cortical perfusion was monitored using the LSCI system. First, mice were anesthetized with 3% isoflurane until they were unresponsive to the tail pinch test, and then maintained at 1.5%. Next, the heads of mice were fixed in a stereotaxic apparatus and the eyes were kept wet with ointment. The scalps were incised longitudinally to fully expose the skull and kept moist with a saline cotton ball. Subsequently, the mice were placed under the LSCI system to acquire a sequence of raw speckle images. The laser speckle temporal contrast analysis method was used to deduce the relative blood flow velocity, and then generate cerebral blood flow (CBF) images. Cortical CBF images on the ipsilateral side were obtained pre stroke (baseline), 30 min after inserting the monofilament, and 10 min after withdrawing the monofilament. Mice without CBF reduction to less than 30% of baseline level after ischaemia or CBF restoration to more than 70% of baseline level after recanalization were excluded from further experimentation.

2D immunostaining

In this work, FITC-conjugated rat anti-Ly6G antibody (127606, Biolegend, dilution 1:50) and Pacific Blue-conjugated rat anti-CD41 antibody (133932, Biolegend, dilution 1:50) was used to label neutrophils and platelets, respectively. And rabbit anti-Glut1 antibody (07-1401, Sigma-Aldrich, dilution 1:500) and Alexa Flour 647-conjugated goat anti-rabbit IgG (A21244, Thermo Fisher Scientific, dilution 1:500) were used to label the blood vessels.

First, 0.2% Triton X-100 in 0.01 M PBS (PBST) was prepared. Then, 100-μm-thick brain slices were blocked with 10% goat serum in 0.2% PBST for 1 h and incubated with the primary antibody and following the secondary antibody diluted with 5% goat serum in 0.2% PBST for 24 h. Finally, brain slices were washed six times with 0.2% PBST for 30 min. For nuclear staining, the immunostained samples were immersed in 0.01 M PBS containing 1 μg/ml DAPI (Invitrogen, New York, USA) with shaking at room temperature for 2 h.

3D immunostaining

Whole-mount immunostaining was performed as previously described in the iDISCO+ protocol, including methanol pretreatment and immunolabeling protocol.[1]

Methanol pretreatment. First, samples were dehydrated in methanol solutions at increasing concentration gradients (20 vol. %, 40 vol. %, 60 vol. %, 80 vol. %, 100 % and 100 % in dH_2O) for 1 h/step. The dehydrated samples were incubated overnight with 66% dichloromethane in methanol, followed by two washes in 100% methanol for 1 h/wash. Samples were then bleached with 5% H_2O_2 in methanol at 4 °C overnight. After bleaching, samples were rehydrated in methanol solutions at decreasing concentration gradients (60 vol. %, 40 vol. %, 20 vol. % in dH_2O) for 1 h/step, and washed twice in 0.01 M PBS for 2 h. Except for bleaching, other steps are processed at room temperature.

Immunolabeling protocol. After methanol pretreatment, samples were blocked in PBST with 10% goat serum for 2 h, incubated in rabbit anti-αSMA (ab5694, Abcam, dilution 1:300) diluted in PBST with 5% goat serum for 1 d, and washed with PBST for 12 h., Samples were then incubated in Alexa Flour 647-conjugated goat anti-rabbit IgG (A21244, Thermo Fisher Scientific, dilution 1:500) diluted in PBST with 5% goat serum for 1 d, washed with PBST for 12 h, and stored in 0.01 M PBS at 4 °C before clearing and imaging. All steps were processed at room temperature.

Microscopy

The z-stack fluorescence images of brain blocks were acquired with an inverted confocal fluorescence microscope (LSM710, Zeiss, Germany) equipped with a Fluar 10×/0.5 dry objective (W.D. 2.0 mm), a Plan-Apochromat 20×/0.8 dry objective (W.D. 0.55 mm) and a Plan-Apochromat 40×/1.4 oil objective (W.D. 0.13 mm). When using the 10× objective, we chose a pinhole of 40 and an interval of 3 μm (**Figures 1-3, 5B, S3-S6**), when using the 20× objective, we chose a pinhole of 30 and an interval of 0.8 μm (**Figures 4, 5C, 6, S2, S7-S9**), and when using the 40× objective, we chose a pinhole of 20 and an interval of 0.42 μm (**Figure 7**). To analyze the occluded microvessels in **Figures 2F, 5B and S4D**, we imaged an area of approximately 1.0 mm (x) by 1.5 mm (y) by 0.6 mm (z) volume using the 10× objective. To analyze vascular diameter in **Figure 7**, we imaged an area of about approximately 0.5 mm (x) by 0.5 mm (y) by 0.1 mm (z) volume using the 40× objective.

Whole-brain vessel images in **Figure S4A** were acquired with a light-sheet microscope (LiTone XL, Light Innovation technology, Hongkong, China) equipped with a 4×/0.25 dry objective (W.D. 25 mm) and a 10×/0.5 objective (W.D. 5 mm). For imaging whole brains, we chose the 4× objective and an interval of 5 μm. For imaging brain regions, we chose the 10× objective and an interval of 3 μm.

Image processing

ImageJ and Imaris software were used to visualize and analyze 2D and 3D images. The maximum intensity projection (MIP) of the z-stack was performed with sequential images in the ImageJ software. 3D images and movies were reconstructed and captured with the Imaris software.

To extract occlude microvessels, we used the ImageJ software to process the z-stack images (**Figure S5**). First, the "Binary" tool was used to calculate the threshold value of each image in the image stack. Next, the binary image of “functional vessels” (the second tomato lectin) was subtracted from the binary image of “existing vessels” (the first tomato lectin) using the “Image calculator” tool to obtain the resulting image, and this resulting image was processed using the “median filter algorithm” in “Filter” tool. Using “And” operation of the “Image calculator” tool, the original image stack of “existing vessels” and the filtered resulting image were calculated to obtain the image stack of “occluded vessels”. In addition, the background signals of the image stacks were removed by “And” operation between the original images and the binary images. In the end, the image stacks of “functional vessels”, “existing vessels”, and “occluded vessels” were

merged using the "Color" tool to generate a new three-channel image stack.

To calculate the vascular diameter, we used the ImageJ software to segment the blood vessel images. First, the "Binary" tool was used to calculate the threshold value of each image in the image stack. Then, the "Filter" tool was used to remove the background signal in the binary images. At last, the "Morphology" tool was used to fill the holes in the signal of the blood vessels.

Quantifications

Changes in cortical perfusion. The relative blood flow velocity in cortical branch of the middle cerebral artery was measured. The relative blood flow velocity before tMCAo was used as the baseline, and the ratio of blood flow to baseline was used to determine changes in cortical perfusion during and after tMCAo. Each value was obtained by averaging values for the three regions.

The degree of microvascular obstruction. The ratio of vascular length of "functional vessels" to "existing vessels" and the relative volume of "occluded vessels" were both used to quantify the degree of microvascular obstruction. The Imaris software was used for quantification of vascular length and vascular volume. To quantify vascular length, the "filament" tool was used to trace the blood vessels automatically and the total length of the filament was utilized as the total vascular length. The length of "functional vessels" are divided by the length of "existing vessels" to obtain the ratio of vascular length of "functional vessels". To quantify vascular volume, the "surface" tool was used to segment the blood vessels. Surfaces smaller than 1000 μm^3 were removed as clutter, and then the volumes of the total surface in each channel were calculated. The volume of "occluded vessels" was divided by the volume of "Existing vessels" to determine the relative volume of "occluded vessels".

The number of neutrophils. We immunostained neutrophils with the FITC-conjugated anti-Ly6G antibody, stained the nuclear with DAPI, and labeled blood vessels by intravenously injecting tomato lectin. Images in the cortex and the striatum were observed with 30-μm z-projections. Intravascular markers containing FITC and DAPI signals were recorded as the neutrophils. The number of neutrophils in the ipsilateral and contralateral striatum and cortex was counted. Each value was determined by averaging these numbers in the four regions.

The diameter of the capillary. Segmented blood vessel images were imported into the MATLAB software to calculate the vascular diameter. The "Bwskel" function was used to skeletonized the segmented blood vessel images and obtain the centerline of the blood vessels. The "Bwdist" function was used to calculate the distance between the pixels whose signal is "1" to its nearest pixel whose signal is "0" in the segmented blood vessel images. The calculated distances in the centerline represented the radii of the blood vessels.

References

1. Renier N, Adams EL, Kirst C, Wu Z, Azevedo R, Kohl J, et al. Mapping of Brain Activity by Automated Volume Analysis of Immediate Early Genes. Cell. 2016; 165: 1789-802.

Supplementary figures

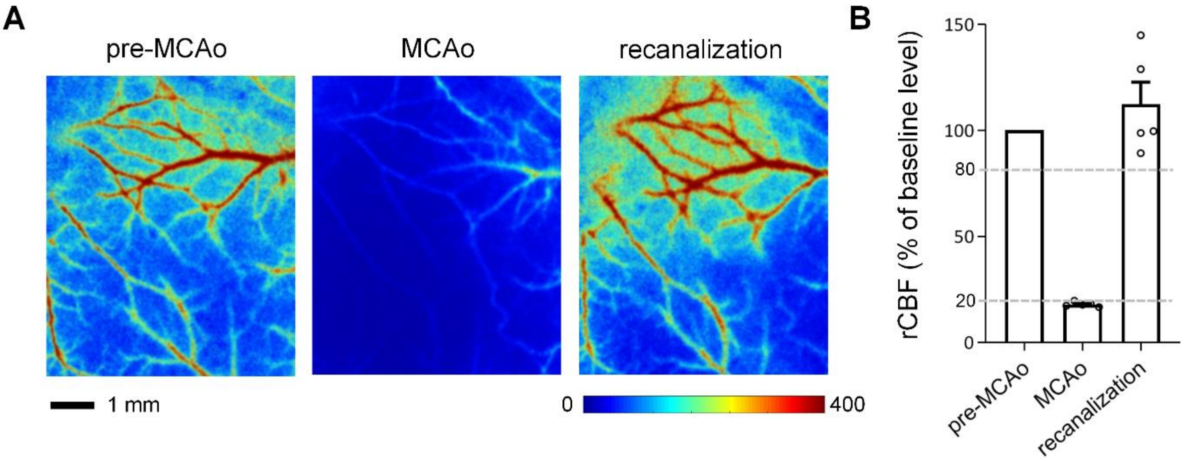

Figure S1. The hemodynamic monitoring on cortical surfaces before, during and after tMCAo (**A**) Representative images showing cerebral blood flow (CBF) pre MCAo (baseline), after MCAo (30min after inserting the filament), and after recanalization (10 min after withdrawing the filament). (**B**) Histogram showing relative CBF (rCBF) of the ischaemic hemisphere during and after tMCAo (n = 5). Data were presented as the mean ± SEM.

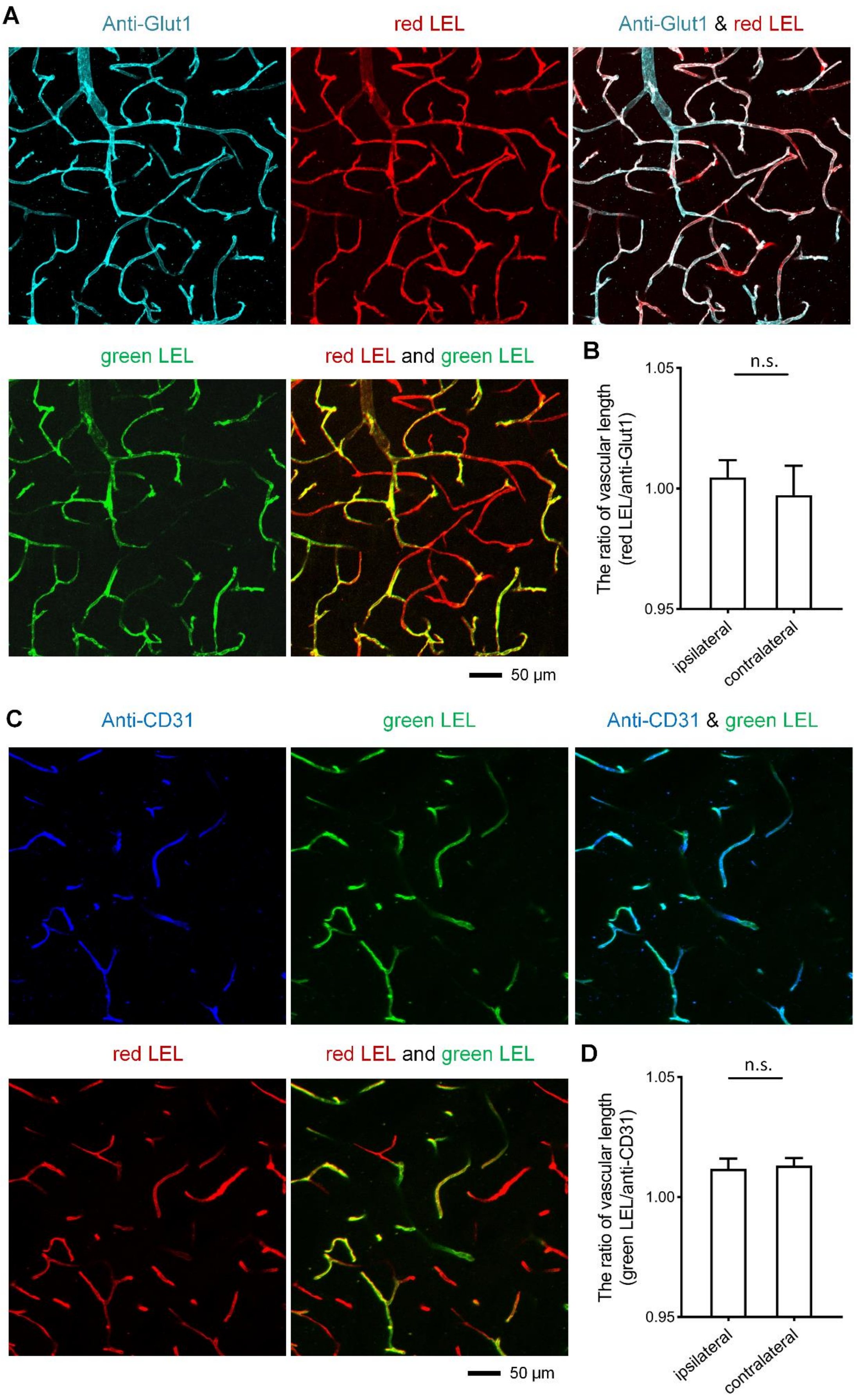
A
Anti-Glut1
red LEL
Anti-Glut1 & red LEL
green LEL
red LEL and green LEL
B
n.s.
1.05
1.00
0.95
The ratio of vascular length
(red LEL/anti-Glut1)
ipsilateral
contralateral
50 µm
C
Anti-CD31
green LEL
Anti-CD31 & green LEL
red LEL
red LEL and green LEL
D
n.s.
1.05
1.00
0.95
The ratio of vascular length
(green LEL/anti-CD31)
ipsilateral
contralateral
50 µm

Figure S2. Verification of vascular labeling by tomato lectin in stroke-injured tissues Mice were subjected to 1 h of ischaemia and 2 h of reperfusion. Red and green LEL were respectively injected into mice through the tail vein before and after I/R. (**A**) Representative fluorescence images showing all blood vessels labeled by red LEL and the anti-Glut1 antibody in the ipsilateral striatum of tMCAO model mice. (**B**) Histogram showing the ratios of the length of the vessels labeled by red LEL to that labeled by the anti-Glut1 antibody in the ipsilateral and contralateral striatum (n = 5). (**C**) Representative fluorescence images showing functional blood vessels labeled by green LEL and the anti-CD31 antibody in the ipsilateral striatum of tMCAO model mice. (**D**) Histogram showing the ratios of the length of the vessels labeled by green LEL to that labeled by the anti-CD31 antibody in the ipsilateral and contralateral striatum (n = 3). Data were presented as the mean ± SD. n.s., $p>0.05$, two-tailed t test.

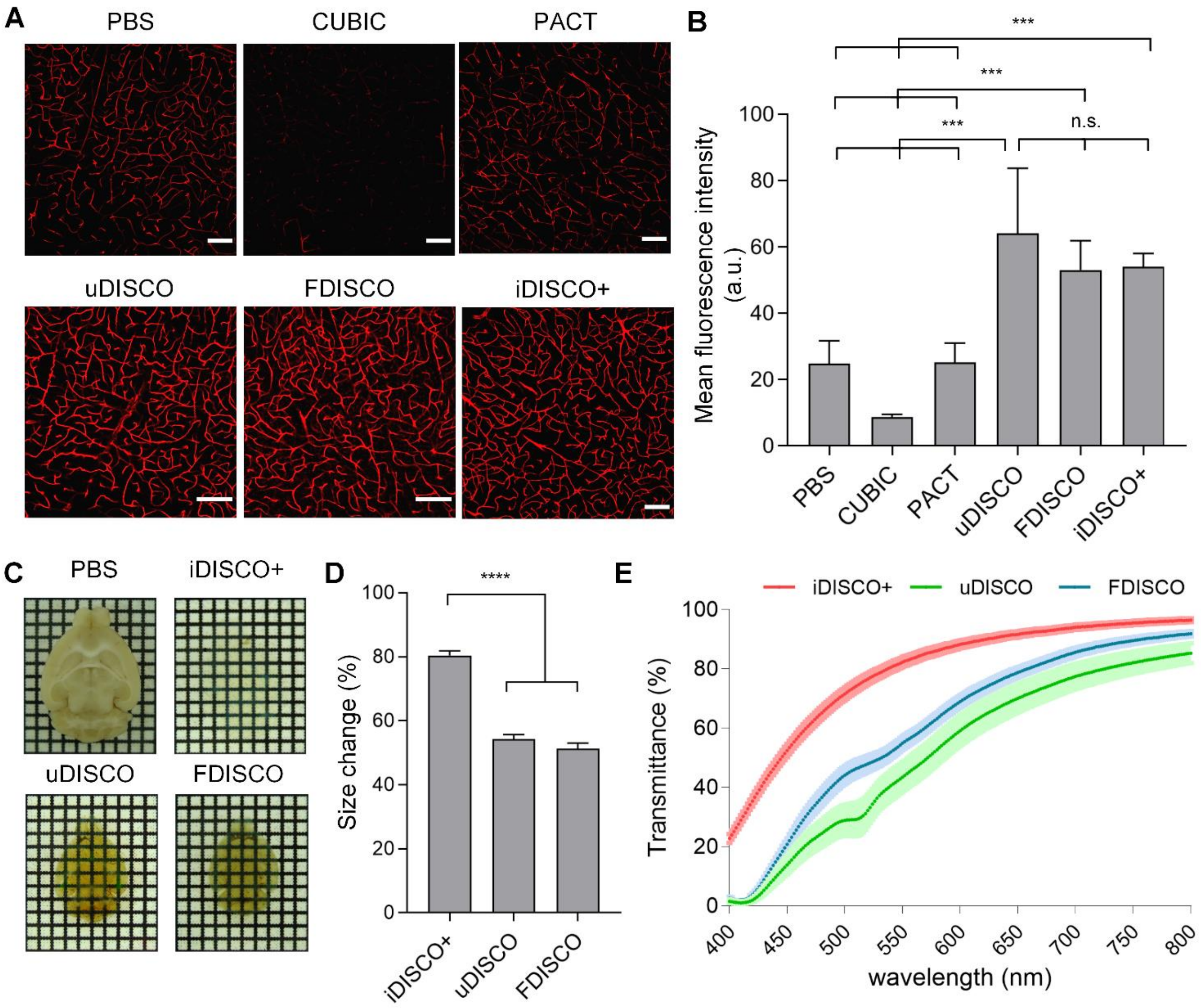

Figure S3. Screening of clearing techniques for imaging microvessels in 3D (**A**) Representative fluorescence images of blood vessels in cleared brain blocks. Scale bar: 100 µm. (**B**) Histogram showing fluorescence signal intensity in cleared brain blocks (n=6). (**C**) Bright-field images of 2-mm-thick brain blocks before and after clearing. Grid size, 1.44 mm × 1.44 mm. (**D**) Histogram showing size change of brain blocks after clearing (n=4). (**E**) Transmittance curves of the brain blocks cleared with iDISCO+, uDISCO, and FDISCO (n=4). Data were presented as the mean ± SD. n.s., p>0.05; ***p<0.001; ****p<0.0001; Brown-Forsythe and Welch ANOVA tests with post hoc Tamhane's T2 multiple comparisons test for each comparison between groups.

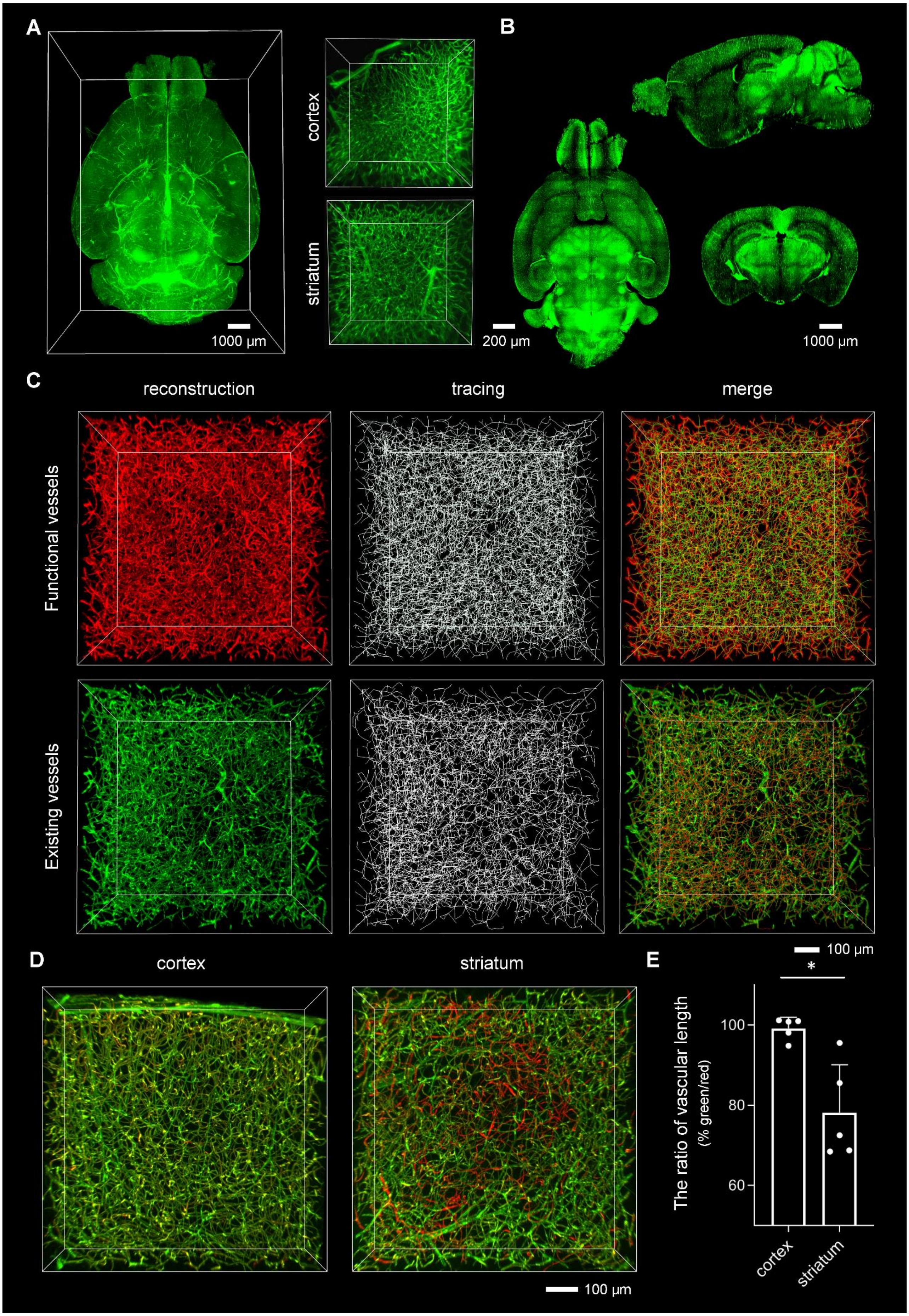
A
cortex
striatum
1000 μm
200 μm
B
1000 μm
C
reconstruction
tracing
merge
Functional vessels
Existing vessels
100 μm
D
cortex
striatum
100 μm
E
*
The ratio of vascular length
(% green/red)
100
80
60
cortex
striatum

Figure S4. Tissue optical clearing assisted 3D analysis of microcirculatory changes after I/R (**A, B**) 3D (**A**) and 2D (**B**) fluorescence images of microvessels in whole mouse brain labeled with tomato lectin, cleared with iDISCO+, and imaged with a light-sheet microscope. (**C**) 3D reconstruction and tracing of microvessels in 1-mm brain slices. Blood vessels were traced using the "filament" tool of the Imaris software to quantify vascular length. The ratio of vascular length of "functional vessels" to "existing vessels" was calculated to obtain the degree of microvascular obstruction. (**D**) 3D fluorescence images of microvessels in the ipsilateral cortex and striatum. The mice were subjected 1 h of ischaemia and 2 h of reperfusion and injected with two different fluorophore-conjugated tomato lectins before and after I/R to identify microvascular obstruction. 1-mm brain slice was cleared with iDISCO+ method and imaged with a confocal microscope. (**E**) Bar graph of the ratio of vascular length showing the degree of microvascular obstruction in the ipsilateral cortex and striatum (n = 5). Data were presented as the mean ± SD. *p<0.05, two-tailed t test.

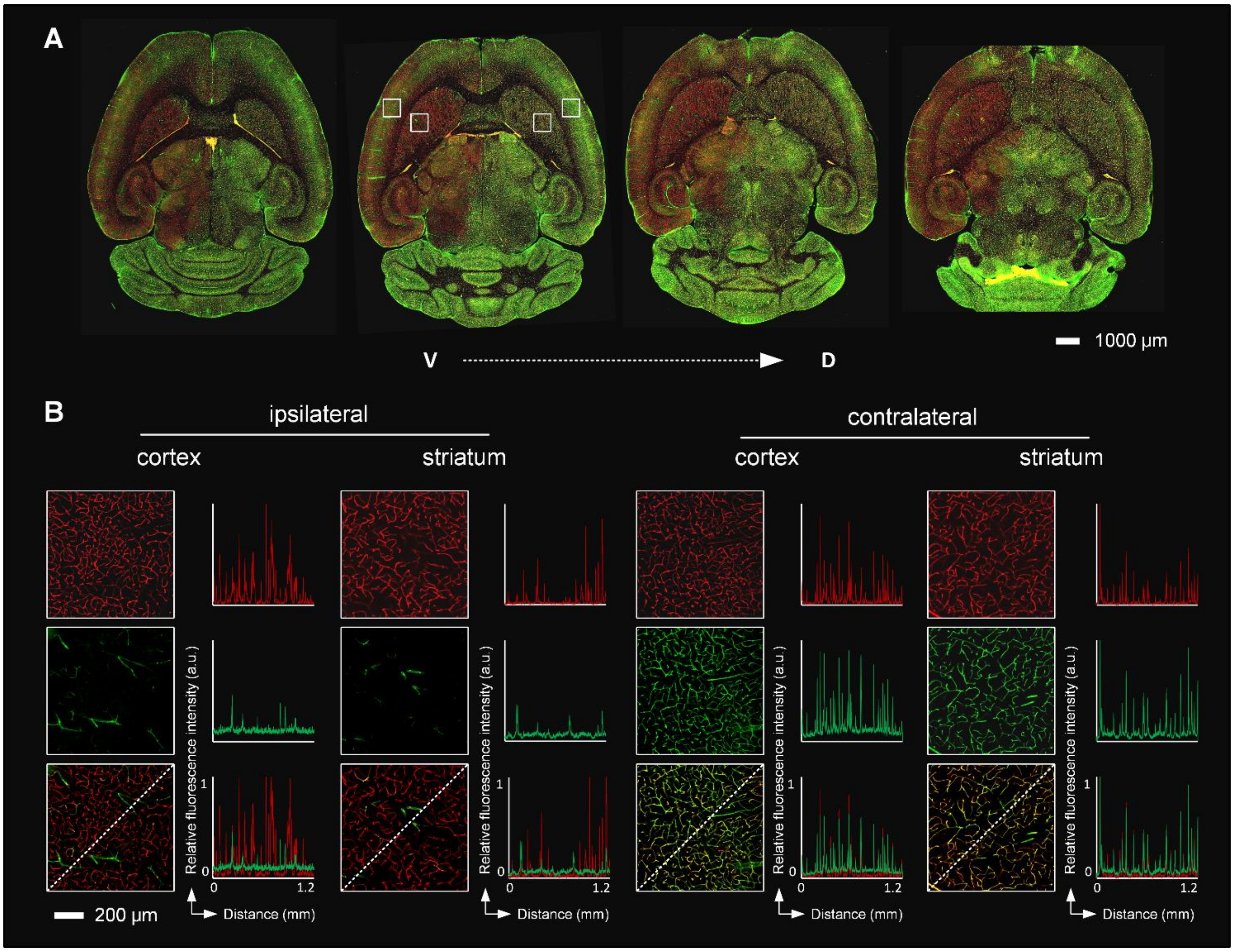

Figure S5. Microcirculatory perfusion after MCAo using a filament (**A**) Representative two-color fluorescent images of 100-μm brain slices in cross section of MCAo model mice showing the ischaemia regions. Red LEL was injected before surgery on the MCAo model, and green LEL was injected 30 min after inserting the filament. The mice were sacrificed 5 min after the second injection. (**B**) Repeated fluorescence images marked with boxes in (**A**) and normalized intensity profiles of marked regions with white dashed lines showing microcirculatory perfusion in the ipsilateral and contralateral cortex and striatum.

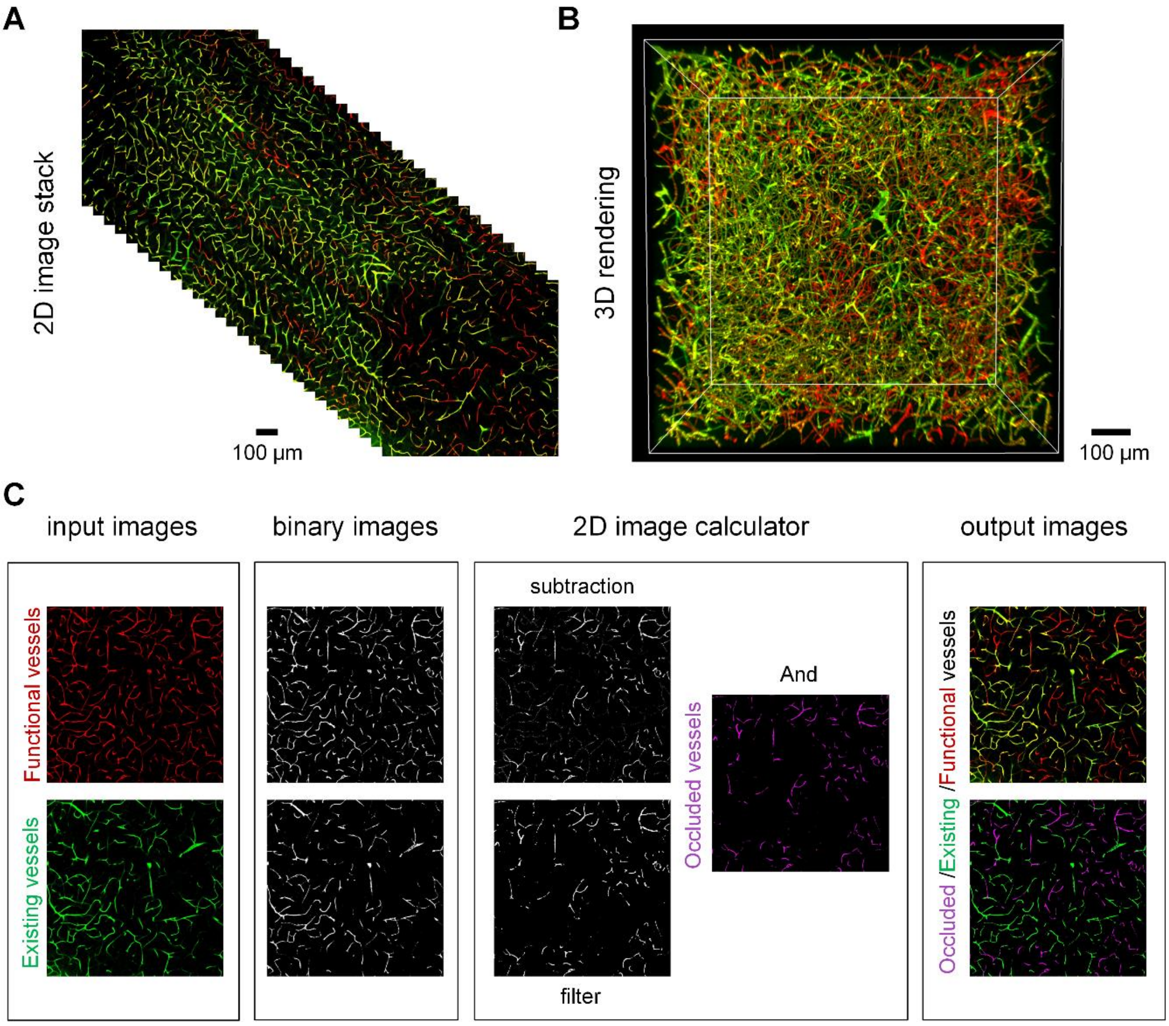

Figure S6. The visualization and analysis of microvessels (**A**) Representative maximum intensity projections (MIPs) showing image stacks across 1-mm-thick brain slices. Each image was 9-µm MIP with 12-µm interval, and the original image was acquired by 3-µm z-step. (**B**) 3D rendering of two-color fluorescence images in (**A**) using the Imaris software. (**C**) Data processing for subtraction of "occluded vessels" using the ImageJ software.

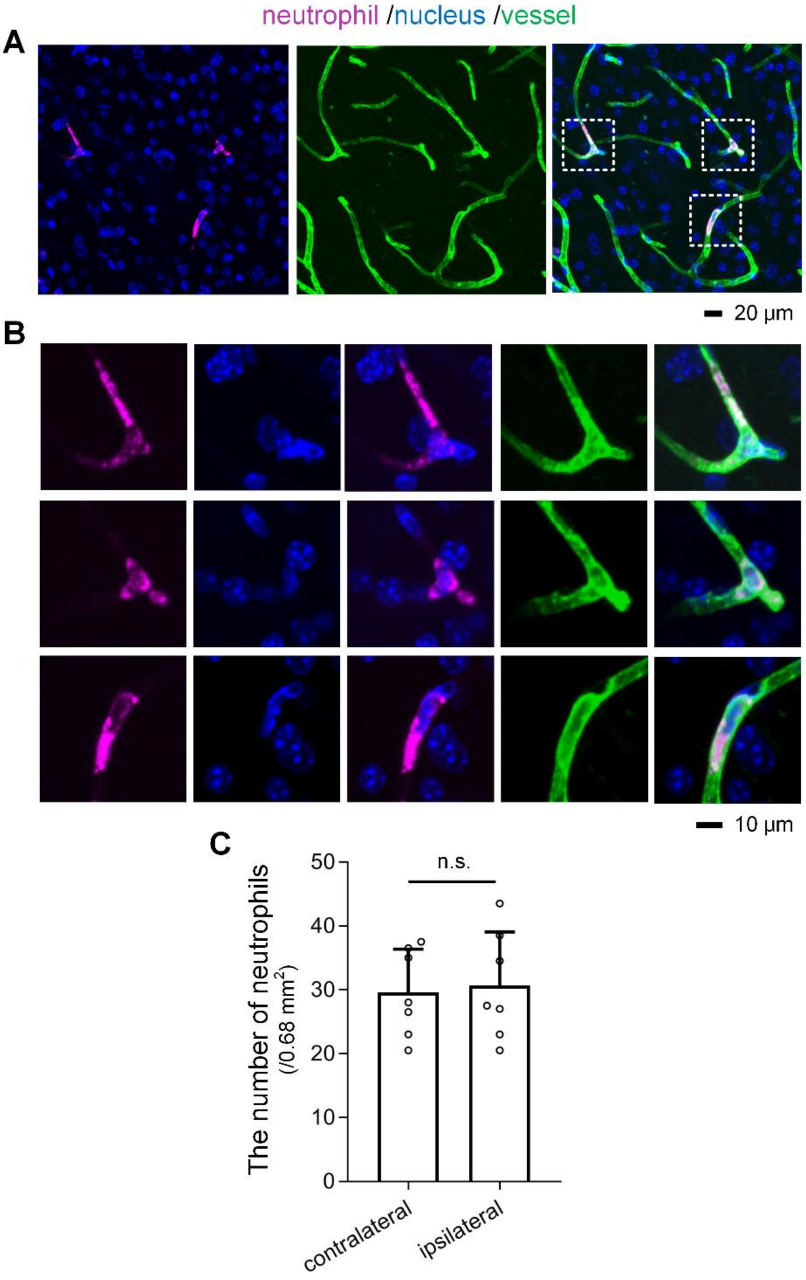

Figure S7. Neutrophils in contralateral cerebral microcirculation (**A, B**) Representative fluorescence images the showing the morphology of neutrophils in contralateral striatum microcirculation. (**B**) Repeated images marked with boxes in (**A**). (**C**) Histogram showing the number of neutrophils in contralateral and ipsilateral cerebral microcirculation (n=7). Data represent the mean ± SD. n.s., $p>0.05$, two-tailed t test.

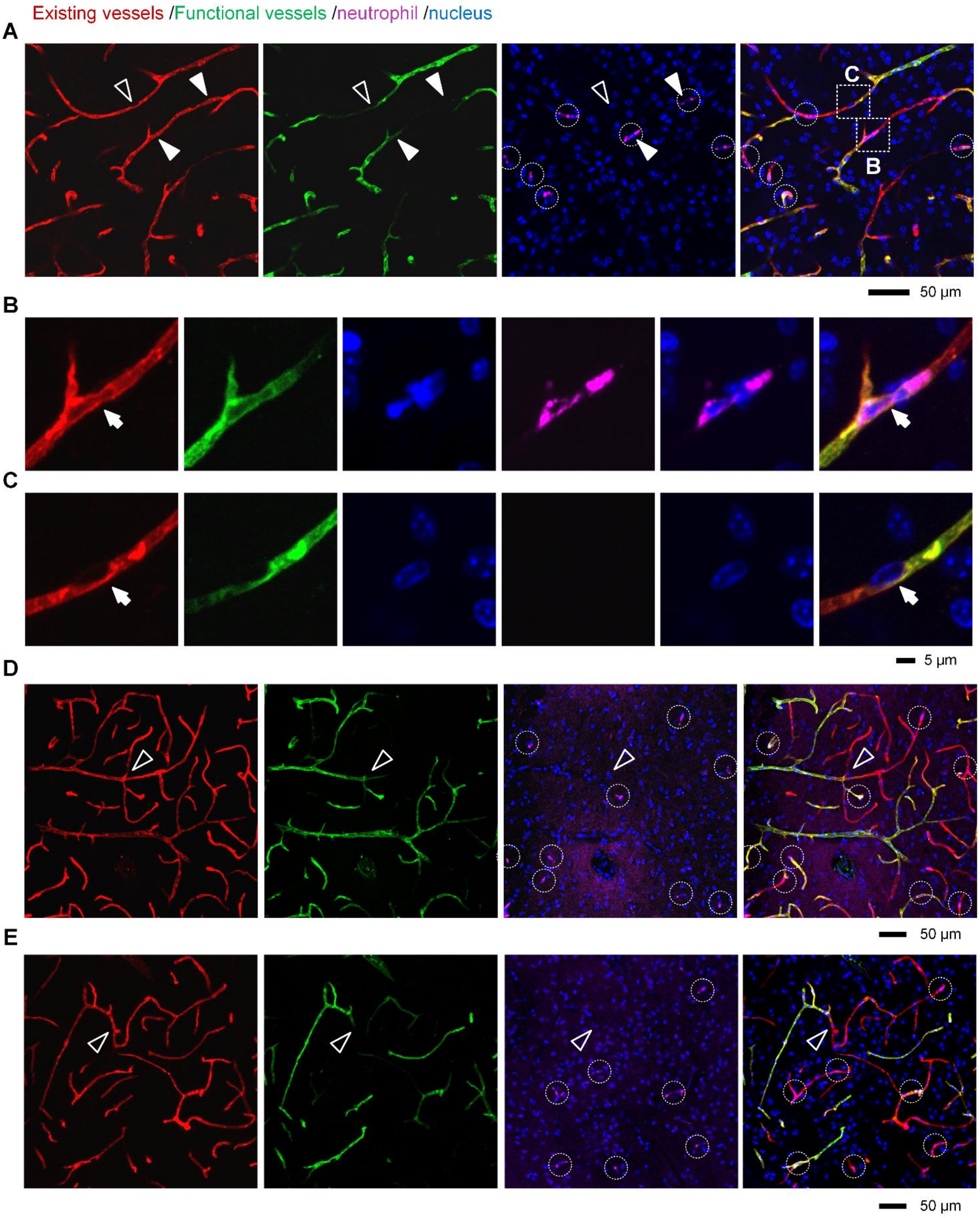

Figure S8. Correlation between microvascular obstruction and neutrophil presence after I/R (**A**) Representative images showing that some capillaries are plugged with neutrophils. (**B-C**) Repeated images marked with boxes in (**A**). (**B**) indicates blockage points with neutrophil plugging, and (**C**) indicates the blockage point without neutrophil plugging. The white arrows indicate blockage points at which the vascular lumen is narrowed. (**B**) also shows that the

narrowed vascular lumen is co-localized with the nucleus, appearing to lock the vessels like a claw. (**D, E**) Representative images showing that some capillaries are plugged without neutrophils. The close arrowheads indicate blockage points with the neutrophils and the open arrowheads indicate blockage points without neutrophils. The white circles enclose neutrophils. red, Existing vessels; green, Functional vessels; purple, Neutrophil; blue, Nucleus.

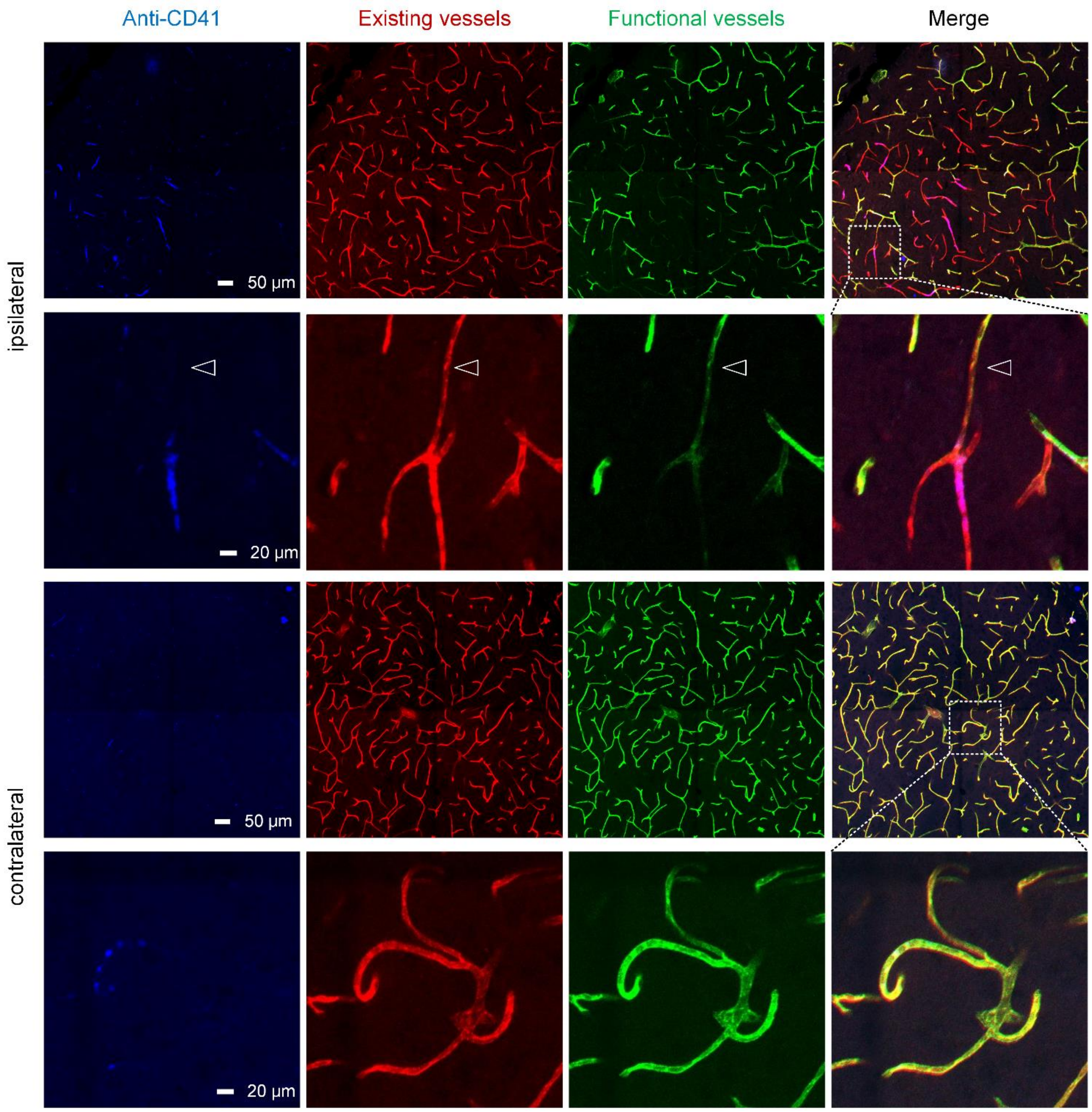

Figure S9. Platelets in cerebral microcirculation after I/R Representative images showing platelet aggregation in ipsilateral and contralateral cerebral microcirculation. Platelets rarely aggregated at the site of blockage. The open arrowhead indicates the blockage point.

Supplementary videos

Video S1. 3D visualization of microvascular obstruction in the ischaemic brain region after I/R. Two different fluorophore-conjugated tomato lectins were injected to mice before and 2 h after tMCAo. 1-mm-thick brain slice was cleared with iDISCO+ method and imaged with confocal microscopy.

Video S2. 3D visualization of microvascular obstruction in whole mouse brain. Whole brain was cleared with iDISCO+ method and imaged with light-sheet microscopy.

Video S3. Vascular tracing for vascular length calculation. Background signals were removed from raw image stacks. The "filament" tool of the Imaris software was used to trace the vessels automatically and the total length of the filament was utilized as the total vascular length.

Video S4. Anti-αSMA antibody helps to determinate the location of blockage points. 1-mm-thick brain slice was immunostained with an anti-αSMA antibody using iDISCO+ method. Magenta-colored vessels indicate arteries, arterioles or large veins labeled with the anti-αSMA antibody.

Video S5. 3D visualization of microvascular obstruction in cleared brain slices from the normal, sham, isotype, and anti-Ly6G groups. Two different fluorophore-conjugated tomato lectins were injected to the mice before and 2 h after tMCAo. 1-mm-thick brain slice was cleared with iDISCO+ method and imaged with confocal microscopy.
